# Step-Detection and Adaptive Step-Length Estimation for Pedestrian Dead-Reckoning at Various Walking Speeds Using a Smartphone

**DOI:** 10.3390/s16091423

**Published:** 2016-09-02

**Authors:** Ngoc-Huynh Ho, Phuc Huu Truong, Gu-Min Jeong

**Affiliations:** School of Electrical Engineering, Kookmin University, 861-1 Jeongnung-dong, Seongbuk-gu, Seoul 136-702, Korea; ngochuynh@kookmin.ac.kr (N.-H.H.); phtruong@kookmin.ac.kr (P.H.T.)

**Keywords:** Weinberg method, PDR, step length, step-detection, FFT filter

## Abstract

We propose a walking distance estimation method based on an adaptive step-length estimator at various walking speeds using a smartphone. First, we apply a fast Fourier transform (FFT)-based smoother on the acceleration data collected by the smartphone to remove the interference signals. Then, we analyze these data using a set of step-detection rules in order to detect walking steps. Using an adaptive estimator, which is based on a model of average step speed, we accurately obtain the walking step length. To evaluate the accuracy of the proposed method, we examine the distance estimation for four different distances and three speed levels. The experimental results show that the proposed method significantly outperforms conventional estimation methods in terms of accuracy.

## 1. Introduction

Pedestrian dead-reckoning (PDR) is extensively studied as an effective approach to obtain pedestrian locations by estimating the distance traveled via handheld inertial sensors [1]. This method can be developed at a low cost for use in conjunction with new services. With the rapid development of microelectromechanical systems (MEMS) in recent years, the demand for pedestrian positioning has been considerably increasing in fields such as navigation systems [2], augmented reality [3], gait analysis [4], and health monitoring [5]. Therefore, it is necessary to accurately detect human walking distances for the determination of pedestrian locations.

Recently, two main types of algorithms for distance estimation have been described. The first type is based on the successive double-integral-based length-step measurement of acceleration. The major drawback of this technique is the error accumulated over the duration of the experiments. This problem can be partially addressed using zero velocity updates (ZUPT). In the second type, researchers applied a verifiable relationship between vertical acceleration and the step length to estimate the distance traveled by a moving subject. Bylemans et al. [6] constructed an empirical solution for step-length estimation using mobile devices equipped on the subject’s body. Another empirical method was based on the correlation of vertical acceleration at the foot of the subject [7,8,9] with the length of the step. Wang et al. [7] developed a wearable sensor module that could be attached to the ankles of the subjects in order to collect the acceleration signal during different walking activities, and then to estimate the walking distance. Indeed, these conventional methods showed good performance in the case of normal walking speed. However, if the user’s speed increased, the estimation errors became excessively large.

Considering these facts, we propose a step-length estimation method based on a previously developed method (namely, the Weinberg method [10]) to improve the estimation accuracy under three speed levels: low, normal, and high. To this end, we define a unit conversion as a function of the step velocity and determine the step length using the Weinberg formula. In the proposed method, we apply the fast Fourier transform (FFT) operation to smooth the obtained accelerations before identifying the step-phase using our step-detection rules. Furthermore, we transform the sensor coordinate system to the world coordinate system for step-length estimation. After the step-detection process, we estimate the step velocity using a linear regression model, and thus determine the length of step. Conclusively, we sum all step lengths during traveling to obtain the walking distance.

The remainder of this paper is structured as follows. First, a review of the conventional methods is presented in Section 2. Section 3 provides our definition of the device’s coordinate system and the ranges of the step levels and presents our proposed method for walking-distance estimation. Section 4 presents the experimental results for four healthy subjects. Finally, the conclusions are presented in Section 5.

## 2. Related Works

In a PDR system, to update the pedestrian’s position, it is necessary to first estimate the length of each step. Numerous PDR estimators were previously proposed for the estimation of the walking distance both indoors [11,12,13,14] and outdoors [14,15]. One of the most renowned distance estimators for smartphones based on a PDR algorithm was presented by Weinberg [10]. According to this method, vertical acceleration was applied to the subject’s hip, in order to determine the walking distance. Then, a unit conversion was used to adjust the estimated walking distance. The proposed Weinberg formula is:

(1) $$L_{1}\approx K_{1}\times \sqrt[{4}]{A_{max}-A_{min}}$$

where $A_{max}$ and $A_{min}$ are the maximum and minimum acceleration values, respectively, measured on the *Z*-axis in a single stride, respectively, and $K_{1}$ is a constant for unit conversion (i.e., feet or meters traveled).

Kim [16] developed an empirical method based on the dependence of average acceleration with each step length during walking. The step length using this method is:

(2) $$L_{2}=K_{2}\times \sqrt[{3}]{\frac{\sum_{i=1}^{M}\left|a_{i}\right|}{N}}$$

where $a_{i}$ is the acceleration measured on sample *i*-th in a single step, *M* is the number of samples corresponding to each step, and $K_{2}$ is a constant for unit conversion.

Finally, Tian [14] designed a tracking system for a handheld device in multi-mode. This method estimates the travel distance based on the height of the subjects and the step frequency. This method’s formula can be expressed as:

(3) $$L_{3}=K_{3}\times h\times \sqrt{f_{s}}$$

where $f_{s}$ is the step frequency measured during the walking experiment, *h* is the height of a certain subject, and $K_{3}$ is a constant for unit conversion.

## 3. Walking Distance Estimation Algorithm

### 3.1. Experimental Setup and Speed Level Definition

**Figure 1:**
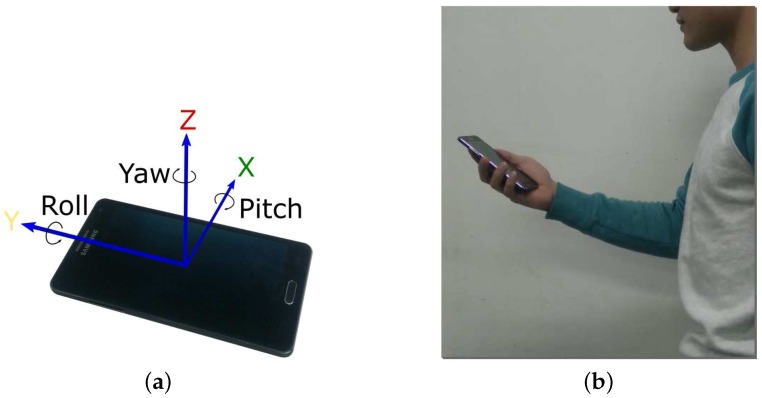
Experimental setup. (**a**) Axes definition; (**b**) Holding mode.

We developed an Android application to collect motion data during the experiments. The lateral direction, longitudinal, and perpendicular directions were denoted as *x*-axis, *y*-axis, and *z*-axis, respectively, as shown in Figure 1a. The experimental setup is illustrated in Figure 1b. Throughout the experiment, the users held the phone in their hand in front of their chest. The participants (four males and four females) were required to walk straight ahead for four different distances: 10, 20, 30, and 40 m. In order to extrapolate the experimental results to different pedestrian groups, the experiment was repeated at three speed levels: low, normal, and high.

**Figure 2:**
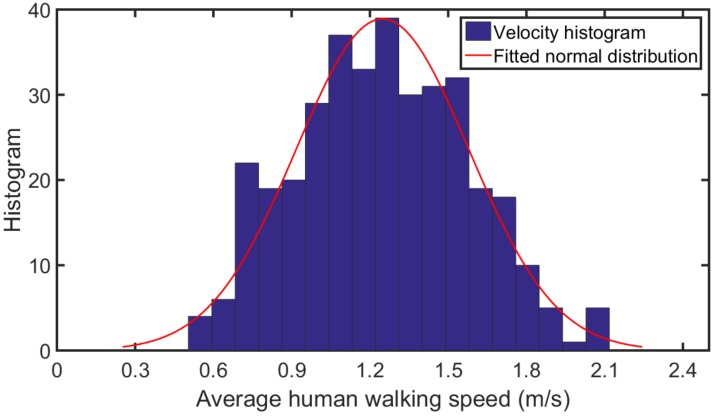
Distribution of average walking speed.

Knoblauch et al. [17] determined the walking speed of young pedestrians (ages 14 to 64) at 1.25 m/s and the walking speed of older pedestrians (ages 65 and older) at 0.97 m/s. According to Bohannon et al. [18], the mean comfortable speed was 1.27 m/s for women and 1.46 m/s for men, whereas the mean maximum speed was 1.75 m/s for women and 2.53 m/s for men. Obaidi et al. [19] concluded that the mean minimum walking speed is 0.8 m/s. It ranges from 1.08 to 1.24 m/s for normal walking, and it reaches 1.81 m/s for fast walking. Tarawneh et al. [20] evaluated the pedestrian speed in Jordan, and found that pedestrian speeds vary from 1.17 to 1.49 m/s. Furthermore, pedestrians between 21 and 30 years old were the fastest group, whereas, those over 65 years were the slowest group. Chandra et al. [21] proposed an average walking speed from 0.97 to 1.36 m/s on various types of facilities (e.g., sidewalk, wide-sidewalk, and precinct). Based on these findings, we defined the speed levels to be used in the present experiments from a normal distribution of average walking velocities as shown in Figure 2. Specifically, we determined the ranges of low, normal, and high speed as $v\leq \bar{v}-\sigma$, $\bar{v}-\sigma <v<\bar{v}+\sigma$, and $v\geq \bar{v}+\sigma$, respectively. Here, $\bar{v}$ and *σ* are the average speed and deviation of human walking speed, respectively. From Figure 2, we set $\bar{v}=1.36$ m/s and σ=0.29. Thus, these walking speed ranges are $\left[0.68,1.07\right]$, $\left[1.08,1.64\right]$, and $\left[1.65,2.05\right]$, respectively, where 0.68 and 2.05 are the minimum and maximum walking speeds in our experiments.

### 3.2. FFT Operation-Based Data Smoothing

We apply FFT [22] to smooth the raw signals for step-detection. Each acceleration signal is represented by a four-dimensional function $f\left(X,Y,Z,t\right)$, where $\left(X,Y,Z\right)$ denotes the space axes, whereas *t* denotes the time axis. We define the first-order finite differences operator matrix (D) as follows:

(4) $$D\left(f\right)=\mathrm{vec}\left(f\left(X,Y,Z,t+1\right)-f\left(X,Y,Z,t\right)\right)$$

where f and $\mathrm{vec}\left(\cdot \right)$ represent the vectorized version of the space–time function $f\left(X,Y,Z,t\right)$ and the vectorization operator, respectively. Our purpose is to obtain a smoothed signal after the inverse FFT (IFFT) process. Therefore, it is necessary to solve error by minimizing the problem in [22]. Then, we obtain the smoothed acceleration function A as follows:

(5) $$A=F^{-1}\left[\frac{F\left[\mu \times a+\rho \times D^{T}\left(u\right)-D^{T}\left(y\right)\right]}{\mu +\rho \times F{\left[\left|D\right|\right]}^{2}}\right]$$

where $F\left[\cdot \right]$ and $F^{-1}\left[\cdot \right]$ are denoted for the FFT and inverse fast Fourier transform (IFFT) functions, respectively. $D^{T}$ is the transpose of D, and *μ*, *ρ* are the input and regularization parameters, respectively. To solve the intermediate variable error, letting $v=D\left(f\right)+\frac{1}{\rho }\times y$, we obtain u as follows:

(6) $$u=\max\left\{\left|v\right|-\frac{1}{\rho },0\right\}\times \mathrm{sign}\left(v\right)$$

In addition, the Lagrange multiplier y was obtained from [22] as follows:

(7) $$y_{l+1}=y_{l}-\rho \times \left(u_{l+1}-D\left(A_{l+1}\right)\right)$$

**Figure 3:**
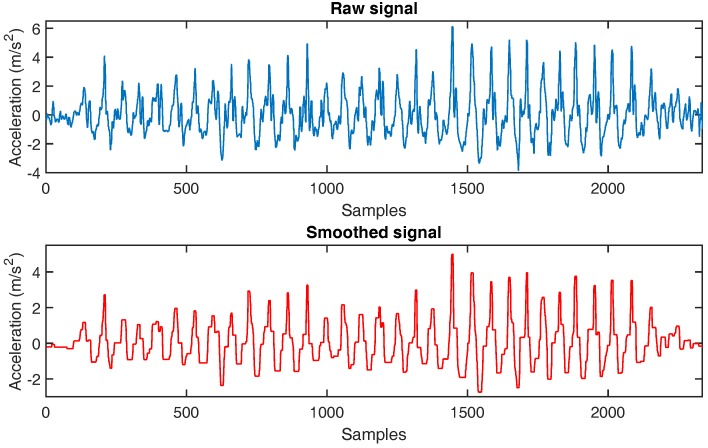
Smoothing of *Z*-axis acceleration.

Algorithm 1 summarizes the FFT operation-based data smoothing method by pseudo-code. Figure 3 shows an example of a raw signal and the corresponding smoothed signal on the *Z*-axis. This figure clarifies that the smoothed signal is less fluctuant than the raw signal. Therefore, we use the smoothed signal to detect the walking step.

**Algorithm 1** Pseudo code for FFT operation-based data smoothing method
Input data: aInput parameter: *μ*Set parameter: *ρ* = 10, l = 100Initialize $f_{0}$ = 0, $u_{0}$ = 0, $y_{0}$ = 0Compute the finite differences operator matrix D and its transpose $D^{T}$***for*** i = 1:l1. Solve A using Equation (5)2. Solve u using Equation (6)3. Update the Lagrange multiplier y using Equation (7)**end**


### 3.3. Step Detection Rules

Step detection is an important problem in PDR that is calculated using the summation of all estimated step lengths. Because of false or missed detection, it can cause substantial errors in total walking distance estimation. Therefore, it is necessary to correctly detect the occurring-step moment. Recently, step detection methods using accelerometers were presented in PDR investigation. Three common methods for acceleration data processing were developed. The first method [23] analyzed the difference of the acceleration data along the vertical dimension (*Z*-axis) while the user was walking. The disadvantage of this method is that the acceleration data could be confusing if the orientation of the accelerometer changed. In the second method [24], the root mean square (RMS) of three axes was used to analyze the feature of the step. The RMS concept used for processing the raw acceleration data is calculated as follows:

(8) $$RMS=\sqrt{{a_{X}}^{2}+{a_{Y}}^{2}+{a_{Z}}^{2}}$$

where $a_{X},a_{Y},\;\mathrm{and}\;a_{Z}$ are the acceleration data of the *X*, *Y*, and *Z* axes, respectively. However, the acceleration sensor did not always maintain a steady state when the user was walking. Therefore, noise tended to increase proportionally with the pedestrian moving speed. The last method is a combination of the acceleration and rotation matrices. In [25], authors used the rotation matrices to transform the acceleration data from the sensor frame to the Earth frame. This method focused on the acceleration data along the vertical dimension. Although the gesture of the accelerometer changed, the data along the vertical orientation maintained normal. Thus, compared with the first method, this method could be more useful in a non-steady state during the movement of the user. However, this method is only valid for low speed movements.

**Figure 4:**
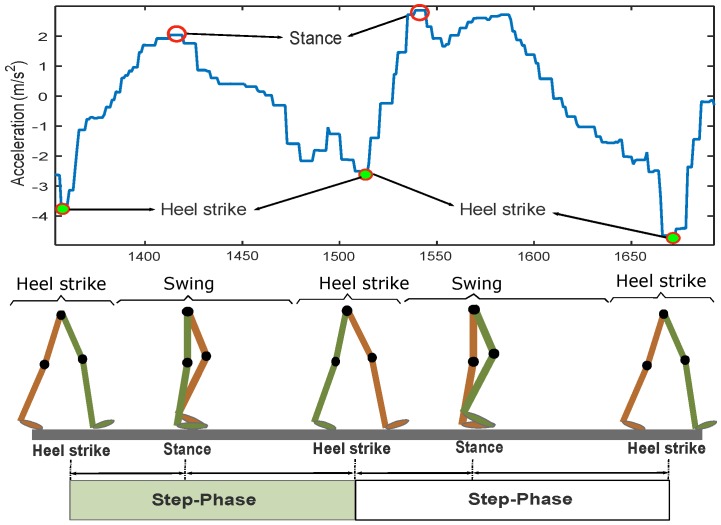
Vertical acceleration based on walking diagram.

In our experiments, the smartphone was tightly held in the users’ hands while they were walking straight ahead. Thus, we can state that the orientation did not significantly change. Based on this analysis, we applied the first method for step detection without considering its drawback. First, we define that a step is composed of a swing phase and a heel strike phase [26]. The swing phase occurs when the reference foot moves forward from the behind of the contralateral leg. The heel strike phase happens when the heel contacts the ground and the waist (the center of gravity) is in its lowest position during the entire step. In the swing phase, the vertical acceleration begins from a minimum valley, then goes to a maximum peak, and finally stops at another minimum valley, which is caused by the next heel strike phase. In other words, the vertical acceleration reaches one maximum peak and one minimum valley within a step. Figure 4 illustrates the pattern of the vertical acceleration in a step.

**Figure 5:**
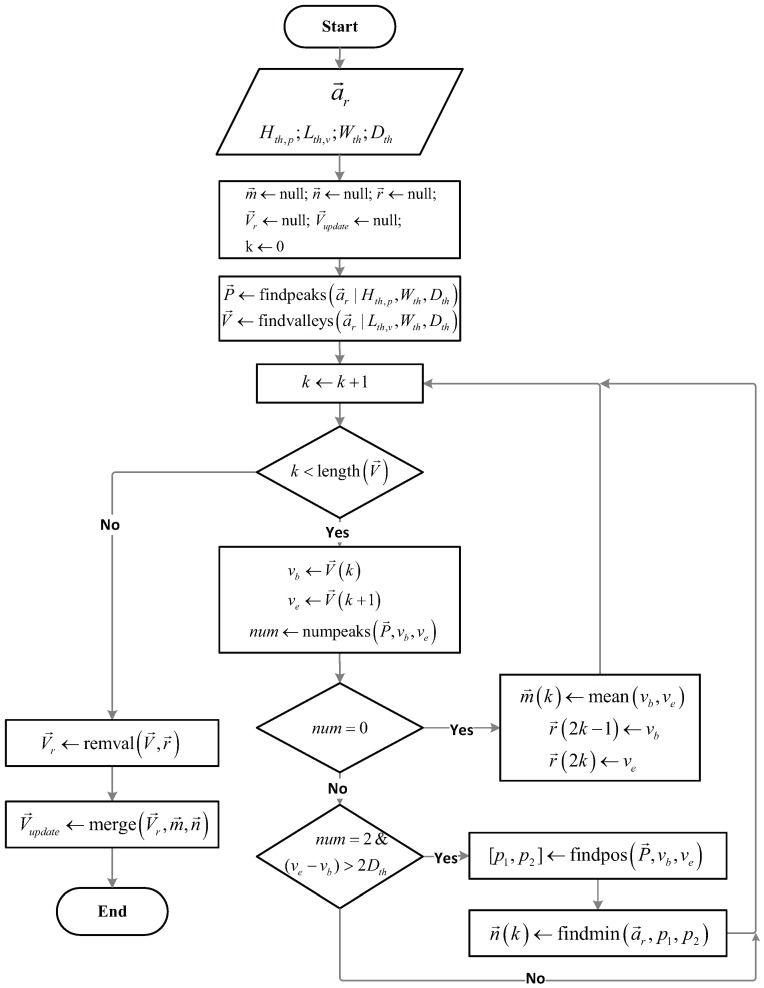
Flowchart of minima-adjustment method.

To detect steps, determination of the maximum peaks (maxima) and minimum valleys (minima) of acceleration data using thresholds can be used. However, this method does not effectively work due to the large variation of the amplitudes of the minima during the heel strike phase. This problem causes no maximum or two maxima between two consecutive minima. On the contrary, the amplitudes of the maxima in the swing phase is less variable. Therefore, it is easy to detect the maxima, but difficult to detect the minima. Considering these facts, we design a new method to fit the minima set. The flow of this method is described in Figure 5.

**Figure 6:**
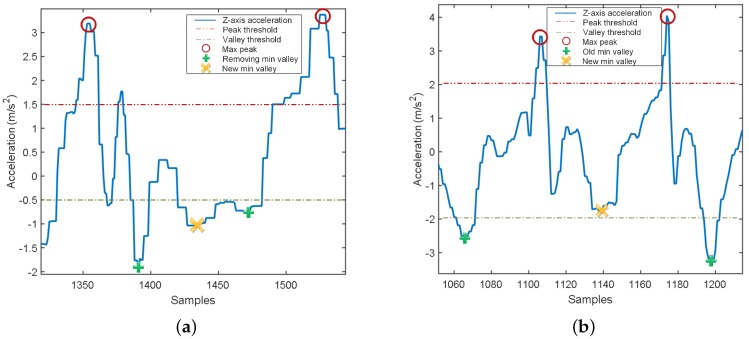
Unexpected minima-set solution in specific cases: (**a**) No maximum; (**b**) Two maxima.

First, we obtain the smoothed-acceleration data ${\vec{a}}_{r}$ using FFT smoother, and assign four thresholds: minimum-peak height ($H_{th,p}$), maximum-valley height ($L_{th,v}$), minimum width ($W_{th}$), and minimum distance ($D_{th}$). The minimum-peak height is used to find peaks greater than $H_{th,p}$, whereas the maximum-valley height is utilized to detect the valleys smaller than $L_{th,v}$. We use the minimum width to locate peaks or valleys whose widths are at least $W_{th}$. The minimum distance is used to find peaks or valleys where the distances between two peaks or two valleys are longer than $D_{th}$. Moreover, we define $\vec{m}$, $\vec{n}$, and $\vec{r}$ as the vectors containing the regulated ($\vec{m}$ and $\vec{n}$) and removed ($\vec{r}$) minima. Based on the thresholds, we determine two sets of maxima $\vec{P}$ and minima $\vec{V}$ using two functions $\mathrm{findpeaks}\left(•\right)$ and $\mathrm{findvalleys}\left(•\right)$, respectively. Then, using the function $\mathrm{numpeaks}\left(•\right)$, we calculate the number of maxima between two consecutive minima $v_{b}$ and $v_{e}$. If there is no maximum in the interval between $v_{b}$ and $v_{e}$, we remove those minima and define a new minimum at the middle of the two removed minima using the function $\mathrm{mean}\left(•\right)$, as shown in Figure 6a. The positions of the removed and regulated minima are stored in $\vec{r}$ and $\vec{m}$, respectively. If there are two maxima between two consecutive minima and the distance of these minima is greater than twice of $D_{th}$, we determine $p_{1}$ and $p_{2}$ positions of those maxima using the function $\mathrm{findpos}\left(•\right)$. In Figure 6b, we locate the lowest valley between $p_{1}$ and $p_{2}$, and set it as a new minimum using the function $\mathrm{findmin}\left(•\right)$. We store the position of the new minima in $\vec{m}$. Then, we remove the unexpected minima in $\vec{r}$ by applying the function $\mathrm{remval}\left(•\right)$ to obtain a new minima set, ${\vec{V}}_{r}$. Finally, using the function $\mathrm{merge}\left(•\right)$, we obtain the updated minima set ${\vec{V}}_{update}$ from ${\vec{V}}_{r}$, $\vec{m}$, and $\vec{n}$ for detection of the step-window and to count the number of steps.

**Figure 7:**
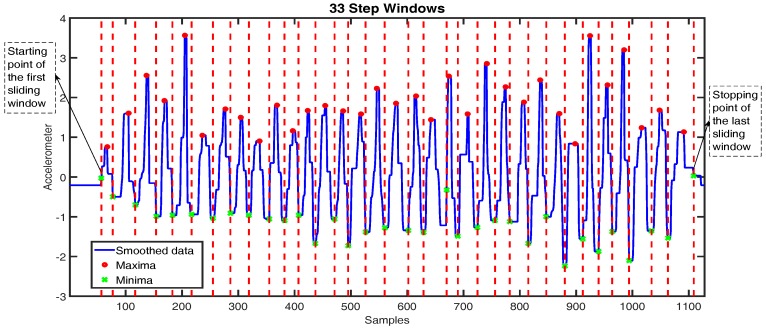
Step detection from smoothed data with maxima and minima.

Figure 7 shows an example of the maxima and minima detection and a step counting result after detecting step windows. We determined the step-sliding window for each step, where the stopping point of the specified window corresponded to the starting point of the next window. We defined the starting point of the first window and the stopping point of the last window as the zero acceleration preceding the first maxima and the zero acceleration succeeding the last maxima, respectively. The duration between two consecutive red dashed vertical lines in Figure 7 indicates the step window size. We detected 33 windows corresponding to 33 walking steps during user movement.

### 3.4. Coordinate System Transformation Using Euler’s Rotation Theorem

Generally, the collected acceleration data are in the smartphone’s coordinates; thus, it is necessary to transform the movement data to the world coordinates. We transformed the three-axis accelerations from the smartphone’s coordinates to the world coordinates using Euler’s rotation theorem [27]. First, we constructed the acceleration and angle vectors from the raw data as follows:

(9) $${\vec{V}}_{acc}=\left[\begin{array}{c} a_{X}\\ a_{Y}\\ a_{Z} \end{array}\right]\;;\;{\vec{V}}_{angle}=\left[\begin{array}{c} \phi \\ \psi \\ \theta \end{array}\right]$$

where, ϕ,ψ,andθ are the yaw, the roll, and the pitch of the phone’s symmetrical axes, respectively. We calculated $T_{c}=\cos\left({\vec{V}}_{angle}\right)$ and $T_{s}=\sin\left({\vec{V}}_{angle}\right)$. Suppose that $T_{c}={\left[\begin{array}{ccc} cx & cy & cz \end{array}\right]}^{T}$ and $T_{s}={\left[\begin{array}{ccc} sx & sy & sz \end{array}\right]}^{T}$, the rotation matrix could be constructed by Euler angles to the Z-Y-X convention as follows:

(10) $$R_{ZYX}=\left[\begin{array}{ccc} cy\times cz & sy\times sx\times cz-sz\times cx & sy\times cx\times cz+sz\times sx\\ cy\times sz & sy\times sx\times sz+cz\times cx & sy\times cx\times sz-cz\times sx\\ -sy & cy\times sx & cy\times cx \end{array}\right]$$

Multiplying the rotation matrix with the acceleration vector, we obtained the rotated acceleration vector as follows:

(11) $$\left[\begin{array}{c} a_{rX}\\ a_{rY}\\ a_{rZ} \end{array}\right]=R_{ZYX}\times {\vec{V}}_{acc}$$

where, $a_{rX},a_{rY},\;\mathrm{and}\;a_{rZ}$ are the rotated acceleration data on three dimensional axes of the world coordinate system.

### 3.5. Adaptive Step Length Based on Unit Conversion

**Figure 8:**
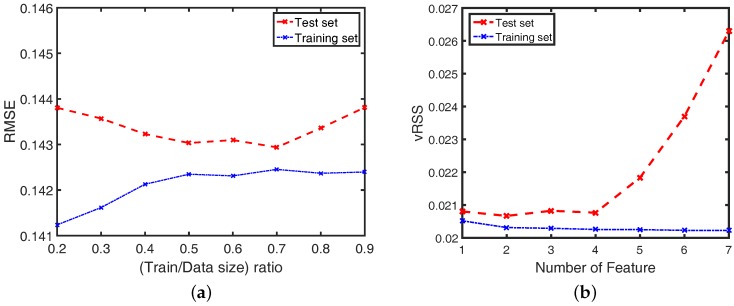
(**a**) RMSE of the actual and estimated velocities for training-set and test-set separation; (**b**) Variance of residual sum of squares (RSS) for degree decision of the polynomial function.

In the proposed method, instead of using a constant *K* for all steps, we derived a *K*-factor as a polynomial function of the average step velocity. To choose the degree of the polynomial function, we applied a *N*-fold Cross-Validation on the dataset. First, we randomly divided the dataset into two sets: a training set and a test set, where the ratio between training set and data adapts from 0.2 to 0.9, and the root mean square errors (RMSE) of the actual velocity and estimated velocity are shown in Figure 8a. From this figure, we decided that the test set included 30% of the samples, whereas the training set composes 70% of the total samples. We examined the variance of the residual sum of squares (vRSS) with *k* first degrees of the polynomial function and repeated this procedure 100 times (*N* = 100). Then, we calculated the mean value of the variances and chose the optimal degrees that minimized the variance in the test set. Figure 8b shows the vRSS for the degree selection of the polynomial function in the test set and the training set. From this figure, we chose two first degrees, ${\bar{v}}_{step}$ and ${\bar{v}}_{step}^{2}$, to train the *K*-value using a linear regression model. The adaptive *K*-value was obtained as follows:

(12) $$K_{vel}=0.68-0.37\times {\bar{v}}_{step}+0.15\times {\bar{v}}_{step}^{2}$$

where ${\bar{v}}_{step}$ was computed as the magnitude of the average velocities on three dimensional axes, *X*, *Y*, and *Z* in each step:

(13) $${\bar{v}}_{step}=\sqrt{{\bar{v}}_{stepX}^{2}+{\bar{v}}_{stepY}^{2}+{\bar{v}}_{stepZ}^{2}}$$

where ${\bar{v}}_{stepX},{\bar{v}}_{stepY},\;\mathrm{and}\;{\bar{v}}_{stepZ}$ were obtained using the accelerometer double-integral formulas:

(14) $$\begin{array}{c} {\bar{v}}_{stepX}=\mathrm{mean}\left(\int a_{r\_stepX}\left(t\right)dt\right)\\ {\bar{v}}_{stepY}=\mathrm{mean}\left(\int a_{r\_stepY}\left(t\right)dt\right)\\ {\bar{v}}_{stepZ}=\mathrm{mean}\left(\int a_{r\_stepZ}\left(t\right)dt\right) \end{array}$$

where $a_{r\_stepX},a_{r\_stepY},\;\mathrm{and}\;a_{r\_stepZ}$ were the acceleration on three dimensional axes of the world coordinate system. The concept of acceleration transformation from the device coordinate system to the world coordinate system is described in Section 3.4. Substituting Equation (12) into Equation (1), for all cases of low, normal, and high speed, we obtained the step length formula as:

(15) $$L_{step}=K_{vel}\times \sqrt[{4}]{A_{max}-A_{min}}$$

Denoting *N* as the number of steps walked during the experiment, we established the traveled distance *D* by summing all the adaptive step lengths as follows:

(16) $$D=\sum_{i=1}^{N}L_{step}\left(i\right)$$

## 4. Experimental Results

**Figure 9:**
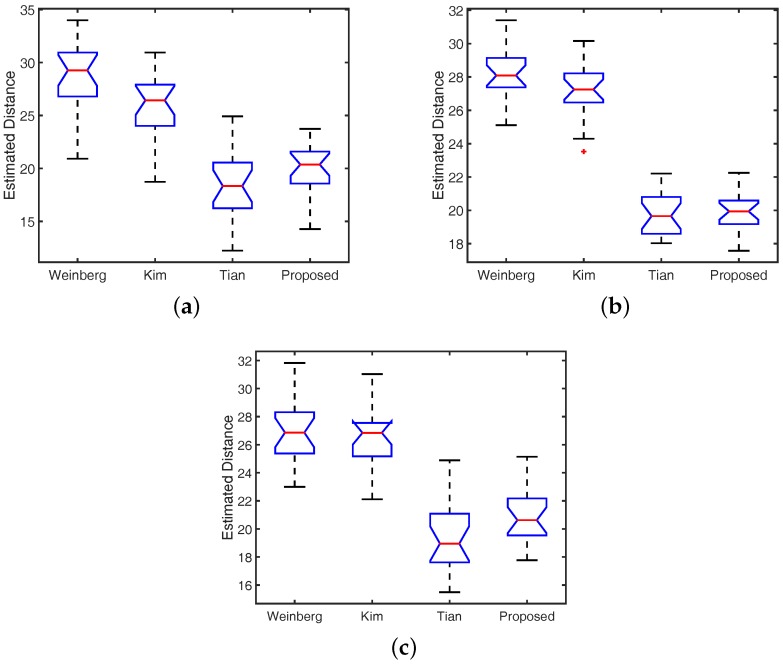
Comparison of the distance estimation methods. (**a**) Slow Walking; (**b**) Normal Walking; (**c**) Fast Walking.

In our experiments, we conducted the above-mentioned walking experiments on eight participants (four males and four females). During this experiment, each person conducted walking experiments covering four distances (10, 20, 30, and 40 m) at three different speed levels (low, normal, and high). The range of walking speeds estimated using Equation (13) was from 0.68 to 2.05 m/s. Figure 9 shows a comparison of the walking distance estimators in the cases of low, normal, and high walking speeds. The vertical axis corresponds to the estimated distance. To illustrate estimating results, we plot the maximum and minimum values, the lower and upper quartiles, and the median of the estimated distance for each estimator. Our proposed estimator provided a better performance than other methods in all speed levels, where the mean estimated distance (the red line) of the proposed method approximates the actual distance (20 m).

Table 1 shows the averages of the estimated walking speeds and step lengths of the proposed method for the four travel distances covered at the three different walking-speed levels. In addition, the table presents the standard deviation (Std) of the average velocity in each distance at each speed level. The last column presents the average results of four indicated distances. Based on these results, the users’ step lengths increase as their speed increases.

Table 2 shows the estimation error (in percentage) and the standard deviation (in meters) results for each walking distance at each speed level of four estimators. The average error column presents the mean error of three speed levels. The results indicate that in all cases, the proposed method performed better than the reference methods. Particularly, Weinnberg [10] and Kim [16] methods acquired high error rates with an average of about 20%, whereas, Tian [14] method obtained better results, where the error rate was about 13%. The proposed method significantly reduced the distance estimation error to 5%. Unlike those methods [10,14,16] which use a constant *K*-value in all the steps for all experimental observations, the proposed method used a velocity-based *K*-value estimator to determine the correlative step length. Therefore, the proposed method can obtain better accuracy of distance estimation for various speed levels.

## 5. Conclusions

This paper proposes a walking-distance estimation method for PDR. In particular, we introduced a new step detection algorithm and a step length estimator. By estimating the step velocity, we defined the unit conversion for each step phase in the step-length estimation process. Then, we determined the total distance during walking by summing all the step lengths. This technique improved the performance of the distance estimator for pedestrian navigation. In the near future, we will investigate the case of walking with a smartphone, such as pedestrians holding the smartphone in their hands and swinging their arms, or putting the smartphone in their pocket during walking. Moreover, we plan to test the estimation method for other types of locomotion, such as running or jogging.

## Figures and Tables

**Table 1:** Speed information and step-length estimation.

Speed Level		10 m	20 m	30 m	40 m	Average
**Low**	Mean ± Std (m/s)	0.94 ± 0.06	0.93 ± 0.04	0.93 ± 0.03	0.95 ± 0.02	**0.937 ± 0.040**
	Step length (m)	0.57	0.60	0.62	0.59	**0.595**
**Normal**	Mean ± Std (m/s)	1.35 ± 0.03	1.36 ± 0.04	1.37 ± 0.04	1.36 ± 0.04	**1.360 ± 0.037**
	Step length (m)	0.70	0.67	0.70	0.69	**0.690**
**High**	Mean ± Std (m/s)	1.72 ± 0.11	1.70 ± 0.04	1.69 ± 0.04	1.69 ± 0.04	**1.700 ± 0.065**
	Step length (m)	0.81	0.82	0.78	0.78	**0.797**

**Table 2:** Estimation errors and standard deviations of walking distance estimators.

Method	Distance (m)	Low Speed		Normal Speed		High Speed		Average Error (%)
		Error (%)	Std (m)	Error (%)	Std (m)	Error (%)	Std (m)		
Weinberg [10]	10	16.95	1.16	17.94	0.69	24.03	0.67	**19.64**
	20	21.44	1.87	20.67	1.72	25.62	1.27	**22.57**
	30	16.71	2.45	21.56	1.97	26.42	1.91	**21.56**
	40	19.00	2.64	22.01	2.81	27.61	2.45	**22.87**
Kim et al. [16]	10	22.59	1.22	18.54	0.74	20.17	0.82	**20.43**
	20	26.05	1.87	20.53	2.03	22.55	1.42	**23.04**
	30	22.10	2.01	21.55	2.57	23.30	2.50	**22.31**
	40	23.42	2.56	22.32	3.13	24.73	2.84	**23.69**
Tian et al. [14]	10	14.36	1.91	10.86	0.99	7.25	0.91	**10.82**
	20	15.48	3.64	8.16	2.42	10.53	2.39	**11.24**
	30	11.34	3.60	11.03	3.12	11.74	3.45	**11.37**
	40	19.48	4.83	13.15	3.26	20.13	6.72	**17.58**
Proposed Method	10	4.76	1.31	4.44	0.87	5.06	1.13	**4.89**
	20	4.51	1.86	4.78	1.84	4.15	1.69	**4.48**
	30	4.52	2.91	4.51	2.41	4.63	2.43	**4.55**
	40	4.32	2.15	4.46	3.22	3.81	2.94	**4.19**
